# Evaluating the quality of feed fats and oils and their effects on pig growth performance

**DOI:** 10.1186/s40104-015-0005-4

**Published:** 2015-03-21

**Authors:** Gerald C Shurson, Brian J Kerr, Andrea R Hanson

**Affiliations:** Department of Animal Science, University of Minnesota, St. Paul, MN 55018 USA; USDA-ARS-National Laboratory for Agriculture and the Environment, Ames, IA 50011 USA; SVC-Research, St. Peter, MN 56082 USA

**Keywords:** Growth, Indicative tests, Lipids, Metabolic oxidation, Peroxidation, Pigs

## Abstract

Feed fats and oils provide significant amounts of energy to swine diets, but there is large variation in composition, quality, feeding value, and price among sources. Common measures of lipid quality include moisture, insolubles, and unsaponifiables (MIU), titer, and free fatty acid content, but provide limited information regarding their feeding value. Lipid peroxidation is an important quality factor related to animal growth performance and health, but maximum tolerable limits in various lipids have not been established. Several indicative assays can be used to detect the presence of various peroxidation compounds, but due to the complexity and numerous compounds produced and degraded during peroxidation process, no single method can adequately determine the extent of peroxidation. Until further information is available, using a combination of peroxide value, thiobarbituric acid reactive substances (TBARS), and anisidine value appear to provide a reasonable assessment of the extent of peroxidation in a lipid at a reasonable cost. However, fatty acid composition of the lipid being evaluated should be considered when selecting specific assays. Predictive tests can also be used to estimate the stability or susceptibility of lipids to peroxidation and include active oxygen method, oil stability index, and oxygen bomb method. A review of 16 published studies with pigs has shown an average decrease of 11.4% in growth rate, 8.8% feed intake fed isocaloric diets containing peroxidized lipids compared to diets containing unperoxidized lipids of the same source. Furthermore, serum vitamin E content was generally reduced and serum TBARS content was increased when peroxidized lipids were fed in these studies, suggesting that feeding peroxidized lipids negatively affects metabolic oxidative status of pigs. However, it is unclear if antioxidants are useful additions to lipids to maintain optimal nutritional value, or if their addition to swine diets is beneficial in overcoming a metabolic oxidative challenge.

## Introduction

Energy is the most expensive component in swine diets, and record high feed costs in recent years have caused nutritionists to focus on optimizing caloric efficiency of feed ingredients used in commercial feeds. As a result, nutritionists need comprehensive, accurate, meaningful, and standardized analytical methods to quantify lipid peroxidation in feed ingredients before they will be able to effectively evaluate the impact of dietary lipid peroxidation on growth and metabolic oxidative status of animals.

Feed lipids and blended lipid products available in the feed ingredient market, vary substantially in fatty acid composition, energy content, quality, and price. Commonly used lipid quality measurements include color, fatty acid profile, free fatty acid (FFA) content, degree of unsaturation or saturation (iodine value -IV; titer), saponification value, and impurities including moisture, insolubles, and unsaponifiables (MIU). These indices are generally used to ensure that the lipid products meet trading specifications, but provide non-specific or no information the extent of lipid peroxidation and relative feeding value. In a recent survey of lipid quality in the Midwest U.S.A., lipids obtained from a local feed mill had a range in total MIU from 0.8 to 3.7%, active oxygen method (AOM) from 8.0 to 332 h, IV from 66.3 to 84.0 g/100 g lipid, peroxide value (PV) from 0.4 to 7.3 mEq/kg, and free fatty acid (FFA) content from 5.8 to 51.6%. These results indicate that there is a wide range in composition and quality of lipids being fed to livestock and poultry. Unfortunately little is known about the relative effects of each lipid quality measure on digestible (DE) and metabolizable energy (ME) content and nutrient utilization of lipids.

Lipid sources that contain high concentrations of polyunsaturated fatty acids (PUFA) are highly susceptible to peroxidation, especially when exposed to heat, light, oxygen, and transition metals during production, processing, and storage [1]. Lipid peroxidation causes degradation of unsaturated fatty acids resulting in a reduction in energy value [2], as well as deleterious effects on animal health, metabolic oxidative status, and growth performance of pigs [3].

Lipid peroxidation is a complex and dynamic process that simultaneously produces and degrades numerous compounds [1]. Although several indicative and predictive assays have been developed and used to measure various peroxidation compounds, there is no single assay that comprehensively characterizes the extent of peroxidation in all lipid sources. As a result, it is difficult to predict potential negative effects from feeding peroxidized lipids on pig growth performance and health. Although some researchers [4-7] have proposed minimum thresholds of dietary peroxidation that causes reduced growth performance, no generally accepted standards have been established.

## Lipid peroxidation

**Figure 1:**
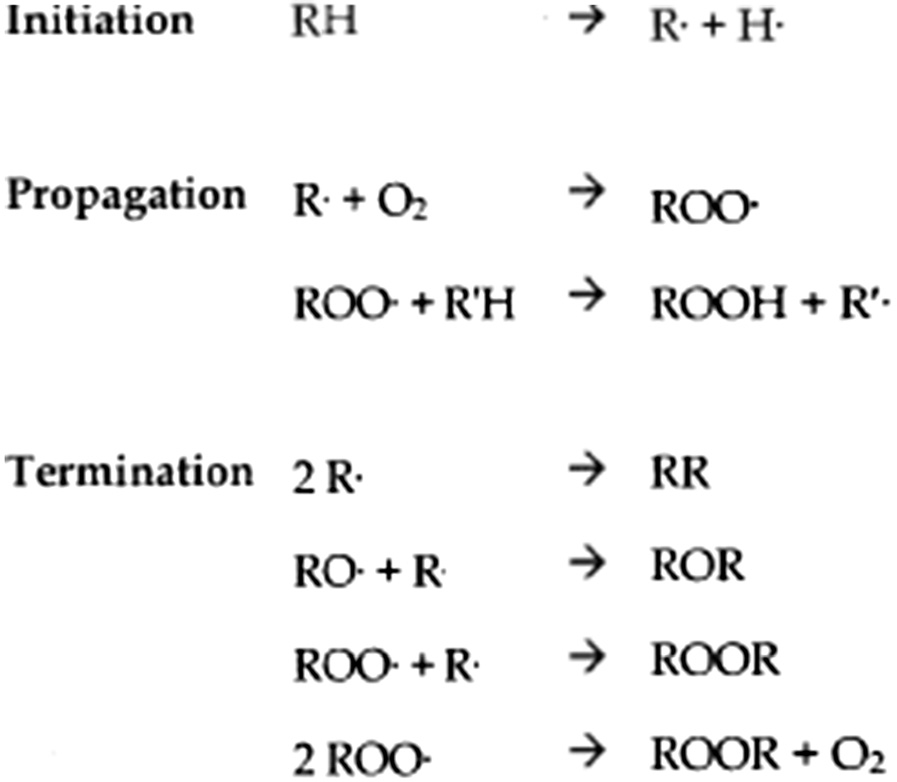
**Free radical induced lipid peroxidation** [12]**.**

**Figure 2:**
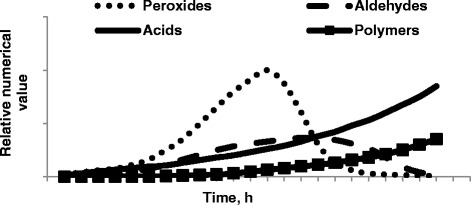
**Simulataneous production and degradation of various peroxidation products occurs during the peroxidation process over time** [8]**.**

Lipid peroxidation is a complex process that is affected by several factors including the degree of saturation, temperature, and the presence of oxygen, transition metals (e.g. Cu and Fe), undissociated salts, water, and other nonlipidic compounds. As shown in Figure 1, lipid peroxidation consists of three phases: initiation, propagation, and termination, with each step “consuming” and producing many compounds [1]. Lipid hydroperoxides initially formed during the lipid peroxidation process not only have the potential to impact lipid quality, but also form secondary and tertiary peroxidation products (aldehydes, ketones, alcohols, hydrocarbons, volatile organic acids, and epoxy compounds) that can have detrimental effects on animal productivity and health. At least 19 volatile compounds are formed during peroxidation of linoleic acid, and these compounds may later be subsequently degraded [1]. However, peroxides and aldehydes that are initially produced are ultimately degraded as peroxidation continues (Figure 2), resulting in underestimation of the extent of peroxidation in excessively peroxidized lipids [8]. Consequently, accurate quantification of the extent of peroxidation of lipids in feed ingredients is challenging because of the complex nature of peroxidation and the numerous compounds produced and degraded during the peroxidation process over time. Therefore, no single method adequately characterizes or predicts lipid peroxidation, and [9] indicates that multiple measures should be used to comprehensively describe the peroxidation status of a lipid.

## Lipid peroxidation measurement

Methods specific for evaluating lipid peroxidation or stability can be divided into indicative and predictive tests. Indicative tests measure specific chemical compounds, or chemically related compounds presentat the time of sampling, and indicate the relative extent that peroxidation has occurred. Predictive tests evaluate the ability of a lipid to withstand peroxidation when exposed to standardized, accelerated conditions to induce peroxidation.

## Indicative tests

A wide variety of indicative tests can be used to quantify lipid peroxidation compounds, but each assay has advantages and disadvantages which must be considered prior to their use. Common indicators of peroxidation in feed fats and oils have been PV, thiobarbituric acid reactive substances (TBARS), and *p*-anisidine value (AnV). However, other measures such as conjugated dienes, TOTOX value, total carbonyls, hexanal value, oxirane value, triacylglycerol dimers and polymers, and total non-elutable material have been occasionally used to assess lipid peroxidation, as well as assays that measure specific peroxidation compounds such as 2,4-decadienal (DDE) and 4-hydroxynonenal (HNE). Unfortunately, peroxidation compounds measured by PV [6,10], TBARS [11], AnV [6], conjugated dienes [12]), total carbonyls [10], and hexanal are produced and subsequently degraded at various stages of the peroxidation process, making interpretation of results difficult and can be misleading. Details of compounds measured and assay limitations have been summarized [13] and presented in Table 1.

**Table 1:** **Compounds measured and assay limitations of indicative tests**

**Indicative measure**	**Peroxidation compounds detected**	**Limitations**
PV	Peroxides and hydroperoxides	Some procedures may be too subjective. Peroxides may be undetectable in lipids exposed to >150°C. Should be used in conjunction with TBARS and AnV when assessing peroxidation.
TBARS	Malondialdehyde	Not specific to malondialdehyde because 2-alkenals, 2,4-alkedienals can react with thiobarbituric acid. Different methodologies are used making inter-laboratory comparisons difficult.
AnV	Aldehydes	Not specific to a particular aldehyde because 2-alkenals, 2,4-alkedienals can react with *p*-anisidine under acidic conditions.
Conjugated dienes	Primary peroxidation compounds formed after a double bond rearrangement in peroxides	Less sensitive compared to PV. Carotenoids are absorbed in the same wavelength range which can cause misleading results.
TOTOX value	Sum of AnV (or TBARS) and 2 × PV. Measures both primary and secondary peroxidation compounds.	Increases the lack of specificity inherent with AnV (or TBARS) and PV.
Carbonyls	Secondary peroxidation compounds including aldehydes and ketones.	Lack of specificity and tendency to be influenced by non-carbonyl compounds.
Hexanal	Specific carbonyl compound formed during the termination phase of peroxidation when linoleic acid (C18:2 n-6) or other ù-6 fatty acids are peroxidized.	Volatile at high temperatures and may provide a misleading indication of extent of peroxidation.
DDE	Specific aldehyde derived from linoleic acid (C18:2 n-6) during peroxidation.	Complicated and expensive assay requiring gas chromatography and mass spectrophotometry.
HNE	α, β-unsaturated lipophilic aldehyde formed during lipid peroxidation of n-6 polyunsaturated fatty acids (i.e. arachidonic and linoleic acid)	Complicated and expensive assay.
Triacylglycerol dimers and polymers	Polymeric compounds formed during the late phases of peroxidation.	Measured with size exclusion chromatography. Limited information on their use in evaluating lipid quality and effects on animal health.
Oxiranes	Cyclic compounds produced during peroxidation.	Assay not specific to oxiranes because it can also detect carbonyls and conjugated dienes.
Non-elutable material	Gas–liquid chromatography procedure that estimates the non-elutable material of a lipid after a correction for glycerol.	Collectively measures most degraded chemical structures of a lipid.

Other more subjective, non-specific indicators include changes in fatty acid profile, decrease in IV [14], increased weight of lipid samples due to oxygen incorporation into lipid hydroperoxides [15], and increased FFA content [11,16]. Unfortunately, these methods are of limited use in practical situations because they require compositional data from the original (unperoxidized) lipid source to determine the magnitude of change that has occurred during peroxidation.

## Predictive tests

Predictive tests evaluate the ability of a lipid to withstand peroxidation when exposed to standardized, accelerated conditions to induce peroxidation. Routinely used predictive tests include the AOM, oil stability index (OSI), and oxygen bomb method (OBM). The AOM has been criticized for the length of time to conduct the assay, particularly for relatively stable lipids [17], modified procedures which makes inter-laboratory comparisons difficult [18], and some have suggested that this method is outdated [19]. Use of OSI offers the advantages compared to AOM because it allows the capability of analyzing multiple samples simultaneously, has a good correlation with AOM [20], and has high inter-laboratory repeatability [18]. The OBM is unique compared with AOM and OSI because it can be conducted on samples without lipid extraction [21], is a faster assay and correlates well (r = 0.89) with AOM, but may be time consuming when evaluating relatively stable samples [22].

## Effect of time, temperature, and lipid source on the production of peroxidation compounds

The effects of lipid composition and peroxidation conditions on the concentration of peroxidation compounds in corn oil, canola oil, poultry fat, or tallow when heated for 72 h at 95°C (slow peroxidation; SO) or heated 7 h at 185°C (rapid peroxidation; RO) with a constant forced airflow rate of 12 L/min have been investigated [11]. Samples were obtained after peroxidation and analyzed for PV, AnV, TBARS, hexanal, DDE, HNE, PUFA, and FFA (Table 2). Free fatty acids increased and PUFA content in all lipid sources decreased after heating. However the magnitude of change was different for each lipid source. For example, the PUFA content declined in both corn oil (9% decrease) and tallow (35% decrease) when exposed to RO conditions. The substantial difference in magnitude of change may be related to the initial PUFA content which is relatively greater in corn oil compared with other lipid sources [9]. Interestingly, PV increased substantially in lipids exposed to SO conditions, but levels increased to a lesser extent under RO conditions. This finding may indicate that high temperatures (i.e. 185°C) expedite the catabolism of peroxides, as suggested by others [19]. The magnitude of change was also greater for SO relative to RO for concentrations of TBARS, hexanal, and DDE, possibly indicating the occurrence of degradation. However, changes in PV, TBARS, hexanal, and DDE concentrations during heating were not monitored. The magnitude of differences under RO conditions compared to SO conditions varied for each lipid source. For example, the hexanal content of SO corn oil increased by 390-fold relative to fresh corn oil, while that of tallow exposed to similar conditions, increased by only 30-fold. This indicates that PUFA content affects the concentration of peroxidation compounds. The magnitude of change relative to fresh lipids was greater for RO compared to SO for AnV and HNE, but only in the vegetable oils. The opposite occurred for tallow or poultry fat. These findings suggest that there is an interactive effect between lipid composition and peroxidation conditions on HNE and AnV, and measurements of lipid peroxidation compounds lead to different responses depending on the fatty acid profile of the lipid, as well as the duration and magnitude of exposure to high temperatures during heating.

**Table 2:** **Indicative measures of lipid peroxidation measures in original lipids (OL) exposed to slow (SO) or rapid peroxidation (RO) conditions** [11]^**1**^

**Items**	**Corn oil**			**Canola oil**			**Poultry fat**			**Tallow**		
	**OL**	**SO**	**RO**	**OL**	**SO**	**RO**	**OL**	**SO**	**RO**	**OL**	**SO**	**RO**
Crude fat, %	99.34	99.36	99.26	99.16	99.50	99.26	95.52	96.42	98.23	98.04	98.68	99.02
Free fatty acids, %	0.28	0.48	0.65	0.36	0.57	0.58	3.62	3.65	3.17	1.99	3.10	2.28
Total MIU^2^	1.00	1.02	1.22	1.01	0.89	0.96	2.24	1.01	1.23	0.78	0.60	0.64
Moisture, %	0.06	0.00	0.06	0.08	0.00	0.00	0.19	0.02	0.07	0.15	0.10	0.07
Insolubles, %	0.02	0.04	0.08	0.02	0.02	0.02	1.08	0.08	0.22	0.22	0.16	0.23
Unsaponifiables, %	0.92	0.98	1.06	0.91	0.87	0.94	0.97	0.93	0.94	0.41	0.34	0.34
Fatty acids, %												
Myristic (14:0)	0.06	0.06	0.07	0.08	0.09	0.08	0.63	0.63	0.65	3.03	3.21	3.29
Palmitic (16:0)	10.76	11.90	12.11	3.95	4.39	4.43	24.69	24.49	24.68	24.50	24.68	25.94
Palmitoleic (16:1)	0.10	0.10	0.12	0.22	0.23	0.23	7.11	7.39	7.19	2.55	2.71	2.55
Stearic (18:0)	1.71	1.91	1.93	1.78	1.93	1.95	5.93	5.62	5.80	21.59	20.00	21.97
Oleic (18:1)	27.70	29.84	29.80	64.57	65.47	66.82	38.07	39.16	39.20	32.03	33.48	30.62
Linoleic (18:2)	57.18	52.73	52.32	17.90	16.51	15.93	18.50	17.59	17.10	2.80	1.83	1.84
Linolenic (18:3)	0.79	0.62	0.63	7.09	5.73	5.01	0.77	0.67	0.69	0.22	0.12	0.11
U:S^3^	6.85	6.01	5.87	15.45	13.72	13.62	2.06	2.11	2.06	0.77	0.80	0.69
Iodine value^4^	125	119	118	105	100	98	73	73	72	35	35	32
Vitamin E, IU/g	0.40	<0.10	<0.10	0.29	<0.10	<0.10	<0.10	<0.10	<0.10	<0.10	<0.10	<0.10
Oxidation products												
PV^5^, mEq/kg	1	151	2	1	239	12	1	57	2	1	29	3
p-Anisidine value^6^	<1	61.4	142.9	1	37.0	154.8	3	88	22	4	120	19
TBARS^7^, μmol/kg	16	225	119	45	968	622	79	151	58	58	61	41
Hexanal, mg/kg	<1	390	83	1	180	59	3	88	22	4	120	19
2,4-decadienal, ppm	72	3,728	1,345	7	1,091	511	30	442	169	47	261	125
HNE^8^, μmol/kg	0	194	594	0	105	221	0	2	0	0	13	6
AOM^9^, mEq/kg	103	575	528	112	419	533	4	298	5	<2	6	446
OSI^10^, h	8.4	<1.0	<1.0	9.2	<1.0	<1.0	24.6	<1.0	<1.0	12.1	<1.0	<1.0

^1^OL: Lipids were stored as received without antioxidants or heating; SO, lipids heated for 72 h at 95°C with constant compressed air flow rate at 12 L/min; RO, lipids heated for 7 h at 185°C with constant compressed air flow rate at 12 L/min.

^2^Total of moisture, insolubles, and unsaponifiables content.

^3^Unsaturated to saturated fatty acid ratio.

^4^Iodine value was calculated as iodine value = (C16:1) × 0.95 + (C18:1) × 0.86 + (C18:2) × 1.732 + (C18:3) × 2.616 (Method Cd 1–25; AOCS, 1998).

^5^PV = peroxide value.

^6^There is no unit for p-anisidine value.

^7^TBARS = thiobarbituric acid reactive substances.

^8^HNE = 4-hydroxynonenal.

^9^AOM = active oxygen method measured as the peroxide value at 20 h of oxidation.

^10^OSI = oil stability index.

As shown in Table 3, correlations among various composition, indicative, and predictive assays for assessing peroxidation in 4 lipids, each with 3 degrees of peroxidation have also been evaluated [11]. However, caution should be used when interpreting these data because significant correlations do not infer a cause and effect relationship due to the potential confounding of lipid source and the peroxidation method used, even though some correlations were found to be significant among various composition and peroxidation measures. For example, moisture, insolubles, and MIU were positively correlated to OSI (r = 0.81, 0.78, and 0.70, respectively). However, in animal fats, the greater OSI was most likely because animal fats have lower concentrations of unsaturated fatty acids and not because they had greater level of moisture and insolubles as shown in Table 2. Peroxide value was positively associated with TBARS, hexanal, and DDE (r = 0.75, 0.76, and 0.61, respectively); AnV was positively correlated with HNE (r = 0.67) and AOM (r = 0.53), but associated negatively with OSI (r = −0.57); TBARS tended to be positively correlated with AOM (r = 0.51); hexanal was positively associated with DDN (r = 0.94) and tended to be positively correlated with AOM (r = 0.57); DDE was positively correlated with HNE (r = 0.49) and AOM (r = 0.65); HNE was positively associated with AOM (r = 0.66); and AOM was negatively correlated with OSI (r = −0.58). The lack of significant correlations among several of the peroxidation measures may be due to the fact that peroxidation reactions occur concurrently during the peroxidation process with primary, secondary and tertiary oxidation products being produced and degraded at different rates depending upon the stage of oxidation [23-25].

**Table 3:** **Correlation matrix among lipid composition and various peroxidation measures** [11]^**1**^

**Items**	**CF**	**FFA**	**MIU**	**Mo**	**In**	**Usap**	**Myr**	**Pal**	**Pmo**	**Ste**	**Ole**	**Lin**	**Linol**	**US**	**IV**	**VE**	**PV**	**AnV**	**TBARS**	**Hex**	**DDE**	**HNE**	**AOM**	**OSI**
CF	1.0	-	-	-	-	-	-	-	-	-	-	-	-	-	-	-	-	-	-	-	-	-	-	-
FFA	−0.81	1.0	-	-	-	-	-	-	-	-	-	-	-	-	-	-	-	-	-	-	-	-	-	-
	0.01																							
MIU	−0.66	NS	1.0	-	-	-	-	-	-	-	-	-	-	-	-	-	-	-	-	-	-	-	-	-
	0.02																							
Mo	−0.57	0.50	NS	1.0	-	-	-	-	-	-	-	-	-	-	-	-	-	-	-	-	-	-	-	-
	0.05	0.10																						
In	−0.77	0.60	0.80	0.77	1.0	-	-	-	-	-	-	-	-	-	-	-	-	-	-	-	-	-	-	-
	0.01	0.04	0.01	0.01																				
Usap	NS	NS	0.58	NS	NS	1.0	-	-	-	-	-	-	-	-	-	-	-	-	-	-	-	-	-	-
			0.05																					
Myr	NS	NS	NS	NS	NS	−0.97	1.0	-	-	-	-	-	-	-	-	-	-	-	-	-	-	-	-	-
						0.01																		
Pal	−0.64	0.89	NS	0.57	0.51	−0.52	0.69	1.0	-	-	-	-	-	-	-	-	-	-	-	-	-	-	-	-
	0.03	0.01		0.05	0.09	0.08	0.01																	
Pmo	−0.86	0.93	NS	NS	0.60	NS	NS	0.77	1.0	-	-	-	-	-	-	-	-	-	-	-	-	-	-	-
	0.01	0.01			0.04			0.01																
Ste	NS	NS	NS	NS	NS	−0.96	0.99	0.71	NS	1.0	-	-	-	-	-	-	-	-	-	-	-	-	-	-
						0.01	0.01	0.01																
Ole	NS	NS	NS	NS	NS	NS	NS	−0.66	NS	NS	1.0	-	-	-	-	-	-	-	-	-	-	-	-	-
								0.02																
Lin	NS	−0.56	NS	NS	NS	0.68	−0.68	NS	NS	−0.68	NS	1.0	-	-	-	-	-	-	-	-	-	-	-	-
		0.06				0.02	0.02			0.01														
Linol	NS	−0.54	NS	NS	NS	NS	NS	−0.80	NS	−0.49	0.95	NS	1.0	-	-	-	-	-	-	-	-	-	-	-
		0.07						0.01		0.10	0.01													
US	0.52	−0.76	NS	−0.51	NS	NS	−0.65	−0.96	−0.63	−0.67	0.83	NS	0.94	1.0	-	-	-	-	-	-	-	-	-	-
	0.09	0.01		0.09			0.02	0.01	0.03	0.02	0.01		0.01											
IV	NS	−0.72	NS	−0.51	NS	0.85	−0.92	−0.79	NS	−0.93	NS	0.85	NS	0.66	1.0		-	-	-	-	-	-	-	-
		0.01		0.09		0.04	0.01	0.01		0.01		0.01		0.02										
VE	NS	−0.47	NS	NS	NS	NS	NS	NS	NS	NS	NS	NS	NS	NS	NS	1.0								
		0.01																						
PV	NS	NS	NS	−0.57	NS	NS	NS	NS	NS	NS	NS	NS	NS	NS	NS	NS	1.0	-	-	-	-	-	-	-
				0.05																				
AnV	NS	NS	NS	NS	NS	NS	NS	NS	NS	NS	NS	NS	NS	NS	NS	NS	NS	1.0	-	-	-	-	-	-
TBARS	NS	NS	NS	−0.58	NS	NS	NS	−0.59	NS	NS	0.70	NS	0.60	0.62	NS	NS	0.75	NS	1.0	-	-	-	-	-
				0.05				0.04			0.01		0.04	0.03			0.01							
Hex	NS	NS	NS	−0.57	NS	NS	NS	NS	NS	NS	NS	NS	NS	NS	0.50	NS	0.76	NS	NS	1.0	-	-	-	-
				0.06											0.10		0.01							
DDE	NS	NS	NS	−0.53	NS	NS	NS	NS	NS	NS	NS	0.56	NS	NS	NS	NS	0.61	NS	NS	0.94	1.0	-	-	-
				0.08								0.06					0.04			0.01				
HNE	NS	NS	NS	NS	NS	NS	NS	NS	NS	NS	NS	0.54	NS	NS	NS	NS	NS	0.67	NS	NS	0.49	1.0	-	-
												0.07						0.02			0.10			
AOM	NS	−0.51	NS	−0.75	NS	NS	NS	NS	−0.50	NS	NS	NS	NS	NS	NS	NS	NS	0.53	0.51	0.57	0.65	0.66	1.0	-
		0.09		0.01					0.10									0.08	0.09	0.06	0.02	0.02		
OSI	−0.60		0.70	0.81	0.78							NS	NS	NS	NS	NS	NS	−0.57			NS	NS	−0.58	1.0
	0.04	NS	0.01	0.01	0.01	NS	NS	NS	NS	NS	NS							0.05	NS	NS			0.05	

^1^Abbreviations: CF = crude fat, FFA = free fatty acids, MIU = moisture, insolubles, and unsaponifiables, Mo = moisture, In = insolubles, Unsap = unsaponifiables, Myr = myristic acid, Pal = palmitic acid, Pmol = palmitoleic acid, Ste = stearic acid, Ole = oleic acid, Lin = linoleic acid, Linol = linolenic acid, US = unsaturated:saturated ratio, IV = iodine value, VE = vitamin E, PV = peroxide value, AnV = p-ansidine value, TBARS = thiobarbituric acid reactive substances,

Hex = hexanal, DDE = 2, 4-decadinal, HNE = 4-hydroxy nonenal, AOM = active oxygen method, and OSI = oil stability index. Top value represents correlation (r value) and bottom value represents significance (*P* value). If no value is given, it was not found to be different at *P* ≤ 0.10 and listed as NS = non-significant.

These results suggest that accurate measurement of the amount of lipid peroxidation may require determining the level of lipid peroxidation at several time intervals using more than one test. A high PV, AnV, as well as concentrations of TBARS, hexanal, DDE, and HNE, along with high AOM and low OSI indicate a high level of lipid peroxidation. It is economical and feasible to use PV as a primary measure of peroxidation if a lipid has been subjected to mild peroxidation because most of the hydroperoxides formed have not been decomposed. However, TBARS and AnV appear to be more accurate and practical measures to use if a lipid has been subjected to a high level of peroxidation because most of the hydroperoxides formed have already been decomposed to yield secondary or tertiary peroxidation compounds. The fatty acid profile of the lipid and the peroxidative conditions to which lipids were exposed (e.g. storage or processing temperature and duration) appear to be important when selecting an indicative assay.

## Effect of lipid peroxidation on energy content and dietary nutrient digestibility

Feeding peroxidized lipids has been shown to reduce energy digestibility in broilers [26,27]. Primary and secondary peroxidation products have been shown to react with amino acids and lipids in the gastrointestinal tract and decrease protein and lipid digestibility in rats [28]. Results from limited published studies have shown inconsistent responses of feeding peroxidized lipids to pigs, which may be related to the accuracy of the indicative tests used to characterize the lipids being evaluated. Increased rancidity of choice white grease (PV of 105 mEq/kg equating to 6.3 mEq/kg diet) decreased feed intake, but fatty acid digestibility was not affected [6]. Dry matter, crude protein, ether extract digestibility, and MEcontent decreased in nursery pigs fed peroxidized fish oil [29]. In contrast, no effect of slow or rapidly peroxidized corn oil, canola oil, poultry fat, and tallow on DE and ME content was observed when these lipids were fed to nursery pigs, nor was there an effect on apparent total tract digestibility of dry matter, gross energy, ether extract, nitrogen, carbon, or sulfur [30].

## Effects of feeding peroxidized lipids on pigand broiler growth performance

No universally accepted practical guidelines for maximal tolerable limits for adding peroxidized lipids to swine and poultry diets have been established. However, some researchers have suggested acceptable peroxidation threshold concentrations using PV as the peroxidation measure [4-7].

Data from studies that measured growth performance of pigs (n = 16 comparisons) and broilers (n = 26 comparisons) fed diets containing peroxidized lipids have been summarized [31]. Only studies evaluating supplemental lipid sources in isocaloric diets were included. Dietary TBARS and PV were obtained from each study, along with response variables including ADG, ADFI, G:F, and circulating concentrations of vitamin E and TBARS. Overall responses for swine and broilers fed diets with peroxidized lipids showed that ADG was 88.8 ± 12.5% (range = 49.8 to 104.6%), ADFI was 92.5 ± 9.0% (range = 67.8 to 109.8%), and G:F was 95.7 ± 7.2% (range = 70.4 to 106.3%) relative to animals fed diets with unperoxidized lipids. The difference in magnitude of change for ADG (11.2%) compared to ADFI (7.5%) suggests that factors in addition to caloric intake contribute to reduced ADG when feeding peroxidized lipids. For swine, ADG was negatively correlated with dietary TBARS content (r = − 0.63), but not PV. For swine and broilers fed peroxidized lipids, serum content of vitamin E was 53.7 ± 26.3% (range = 15.2 to 105.8%, n = 18) and TBARS was 119.7 ± 23.3% (range = 97.0 to 174.8%, n = 12) relative to animals fed unperoxidized lipids, indicating that inclusion of peroxidized lipids in diets contributes to changes in metabolic oxidative status. Historically, PV has been used to assess lipid peroxidation, but TBARS may be a better measure for predicting the effects of lipid peroxidation on growth in swine

## Effects of feeding peroxidized lipids on metabolic oxidative status

Researchers have consistently shown that consumption of peroxidized lipids reduces the antioxidant status of swine [7,32], broilers [33,34], and rats [35] compared with animals fed diets containing unperoxidized lipids. However, it is difficult to relate specific peroxidation indicators and compounds with physiological changes because there is no single measurement or index that completely characterizes metabolic oxidative status of pigs, but several indicators have been commonly used.

Metabolic oxidative status is often characterized by measuring TBARS and antioxidant concentrations in serum, liver, and other tissues. Higher plasma TBARS concentrations, and lower α-tocopherol concentrations were observed in broilers fed peroxidized vegetable oils with a dietary PV of 17.6 meq/kg feed [27]. In swine, feeding peroxidized corn oil with dietary PV of 9 meq/kg feed increased plasma TBARS, and decreased α-tocopherol concentrations in plasma and liver [36], and feeding slow and rapid peroxidized lipids to nursery pigs increased serum TBARS concentrations [7]. However, the lack of an increase in plasma TBARS may be due to the insufficient dietary oxidative challenge (using PV as an indicator of peroxidation in oil and feed), and there may be a threshold level above which feeding peroxidized lipids causes metabolic oxidative stress in pigs.

Increased liver size relative to body weight serves as a biological indicator of toxicity [37]. Research results have shown that feeding diets containing peroxidized lipids result in increased liver size [7,38,39], and this response may be a result of increased synthesis of microsomal enzymes to mitigate toxicity [39]. However, the practical significance of such changes for nutrient metabolism, growth and health of animals is not clear.

Changes in gut barrier function are another indicator of metabolic oxidative status. Intestinal epithelial cells contain relatively high concentrations of PUFA, which are particularly effective in enhancing intestinal epithelia barrier integrity by improving natural resistance [40], but long chain PUFA are susceptible to lipid peroxidation [41]. Peroxidation of PUFA present in intestinal epithelial cell membranes may lead to cell injury, and thus, impair epithelial barrier function due to the disruption of the normal membrane structure and function [42]. Dietary peroxidized lipids induce metabolic oxidative stress in enterocytes [43,44]. There is also histological evidence that the half-life of enterocytes was reduced in broilers fed diets containing peroxidized lipids [45]. However, no effect on intestinal barrier function was observed when diets containing 10% peroxidized corn oil, canola oil, beef tallow, and poultry fat were fed to young pigs [46].

Changes in gene regulation also indicate alterations in lipid metabolism when animals are fed peroxidized lipids. Feeding thermally oxidized lipids to rats [47,48] and pigs [7,49] altered *in vivo* lipid metabolism by activating the peroxisome proliferator-activated receptor α (PPARα) via up-regulation of some target genes in PPARα, such as acyl CoA oxidase, catalase, and carnitine palmitoyltransferase-1. The transcription factor PPARα controls the expression of fatty acid oxidative metabolism in many aspects, including fatty acid uptake through membranes, fatty acid activation, intracellular fatty acid trafficking, fatty acid oxidation, ketogenesis, and triglyceride storage and lipolysis [50]. Some mechanisms regarding these regulatory roles of PPARα in lipid metabolism have been studied, while most of them are still unknown. However, results from a recent study showed that pigs fed thermally oxidized lipids had increased activation of PPARα in the liver, indicating alterations in fatty acid metabolism [7].

## Role of supplemental antioxidants in diets containing peroxidized lipids

Antioxidant chemistry and applications is a complex field of science and this subject has been extensively reviewed [51,52]. Addition of antioxidants (e.g. butylated hydroxyanisole, butylated hydroxytoluene, tocopherol, and ethoxyquin) to human, rodent, livestock, and poultry diets has been evaluated, but their impacts on animal physiological and performance parameters has been inconsistent [36]. Feed conversion was reduced in broilers fed peroxidized poultry fat compared to birds fed unperoxidized poultry fat, but the addition of ethoxyquin to these diets improved feed conversion regardless of lipid peroxidation level [45]. Likewise, supplementation of antioxidants improved growth performance in pigs fed diets containing dried distillers grains with solubles or peroxidized corn oil [36,53]. In contrast, other researchers have shown that supplementation of antioxidants to diets has no effect on growth performance in animals under dietary oxidative stress [36,54-56]. Based on these inconsistent responses, it is unclear if antioxidants are necessary additions to lipids used in animal feed to maintain optimal nutritional value, or if their addition to swine diets are beneficial in overcoming a metabolic oxidative challenge.

## Conclusions

Lipid peroxidation is a dynamic process which produces numerous compounds which have been associated with deleterious effects on animal health, metabolic oxidative status, and growth performance. Consequently, these effects can significantly reduce energy and nutritional efficiency and increase the cost of food animal production. However, accurate measurement of the extent of lipid peroxidation and relationship to animal health and performance is a major obstacle that must be overcome to optimize energy and nutrient utilization efficiency in animal feeds. Currently, there are no universally accepted analytical standards for measuring lipid peroxidation, and various measures are used in different segments of the food, agriculture, and lipid industries. Animal nutritionists have historically assumed that peroxide value and thiobarbituric acid reactive substances assays are reliable indicators of the extent of lipid peroxidation in feed fats and oils. However, a review of the scientific literature and recent studies indicate that the use of PV or TBARS as single indicators do not adequately characterize the extent of lipid peroxidation as it relates to animal performance, and may often provide misleading results. The fatty acid profile of the lipid and the peroxidative conditions to which lipids were exposed (e.g. storage or processing temperature and duration) appear to be important when selecting an indicative assay. Therefore, use of combinations of indicative peroxidation assays that measure compounds at different stages of peroxidation is recommended to provide a more accurate assessment of peroxidation of lipids used in animal feeds, and determine dietary thresholds of peroxidation compounds at which animal growth is impaired. Although the addition of some dietary antioxidants have been shown to improve animal performance when feeding peroxidized lipids, the type of antioxidant and the dietary peroxidation conditions where they are beneficial needs to be defined.

## Abbreviations

ADG: Average daily gain
ADFI: Average daily feed intake
AnV: *p*-Anididine value
AOM: Active oxygen method
DDE: 2,4-decadienal
DE: Digestible energy
FFA: Free fatty acids
G:F: Gain:feed
HNE: 4-hydroxynonenal
IgA: Immunoglobulin A
MDA: Malondialdehyde
ME: Metabolizable energy
MIU: Moisture, insoluble, and unsaponifiables
OSI: Oil stability index
OMB: Oxygen bomb method
PPARα: Peroxisome proliferator-activated receptor
PUFA: Polyunsaturated fatty acids
PV: Peroxide value
RO: Rapid oxidation
SO: Slow oxidation
TBA: Thiobarbituric acid
TBARS: Thiobarbituric acid reactive substances
